# Tumor-targeting *Salmonella typhimurium* A1-R combined with temozolomide regresses malignant melanoma with a BRAF-V600E mutation in a patient-derived orthotopic xenograft (PDOX) model

**DOI:** 10.18632/oncotarget.13231

**Published:** 2016-11-09

**Authors:** Kei Kawaguchi, Kentaro Igarashi, Takashi Murakami, Bartosz Chmielowski, Tasuku Kiyuna, Ming Zhao, Yong Zhang, Arun Singh, Michiaki Unno, Scott D. Nelson, Tara A. Russell, Sarah M. Dry, Yunfeng Li, Fritz C. Eilber, Robert M. Hoffman

**Affiliations:** ^1^ AntiCancer, Inc., San Diego, CA, USA; ^2^ Department of Surgery, University of California, San Diego, CA, USA; ^3^ Department of Surgery, Graduate School of Medicine, Tohoku University, Sendai, Japan; ^4^ Division of Hematology-Oncology, University of California, Los Angeles, CA, USA; ^5^ Department of Pathology, University of California, Los Angeles, CA, USA; ^6^ Division of Surgical Oncology, University of California, Los Angeles, CA, USA

**Keywords:** melanoma, PDOX, nude mice, orthotopic, drug-response

## Abstract

Melanoma is a recalcitrant disease in need of transformative therapuetics. The present study used a patient-derived orthotopic xenograft (PDOX) nude-mouse model of melanoma with a BRAF-V600E mutation to determine the efficacy of temozolomide (TEM) combined with tumor-targeting *Salmonella typhimurium* A1-R. A melanoma obtained from the right chest wall of a patient was grown orthotopically in the right chest wall of nude mice to establish a PDOX model. Two weeks after implantation, 40 PDOX nude mice were divided into 4 groups: G1, control without treatment (*n* = 10); G2, TEM (25 mg/kg, administrated orally daily for 14 consecutive days, *n* = 10); G3, *S. typhimurium* A1-R (5 × 10^7^ CFU/100 μl, i.v., once a week for 2 weeks, *n* = 10); G4, TEM combined with *S. typhimurium* A1-R (25 mg/kg, administrated orally daily for 14 consecutive days and 5 × 10^7^ CFU/100 μl, i.v., once a week for 2 weeks, respectively, *n* = 10). Tumor sizes were measured with calipers twice a week. On day 14 from initiation of treatment, all treatments significantly inhibited tumor growth compared to untreated control (TEM: *p* < 0.0001; *S. typhimurium* A1-R: *p* < 0.0001; TEM combined with *S. typhimurium* A1-R: *p* < 0.0001). TEM combined with *S. typhimurium* A1-R was significantly more effective than either *S. typhimurium* A1-R (*p* = 0.0004) alone or TEM alone (*p* = 0.0017). TEM combined with S. typhimurium A1-R could regress the melanoma in the PDOX model and has important future clinical potential for melanoma patients.

## INTRODUCTION

Melanoma becomes a recalcitrant cancer when it metastasizes to regional lymph nodes, with a 5-year survival rate of 29% and 7% when it metastasizes to organs [1]. Dacarbazine and cisplatinum have been used to treat melanoma with limited efficacy [1–5]. Temozolomide (TEM) is an alkylating agent, had been widely used as a first-line chemotherapy for melanoma but with limited efficacy [1–5]. Although recently-developed immuno-therapy has extended survival to some extent, the 5-year survival rate has not significantly increased [1–5]. There is still no cure for stage III and IV melanoma due to drug resistance, tumor heterogeneity and an immune-suppressed tumor microenvironment [5]. Therefore, more effective approaches to melanoma treatment are needed.

Clinically-relevant mouse models of melanoma could permit evaluation of tailor-made therapy based on the patient-derived tumor. Our laboratory pioneered the patient-derived orthotopic xenograft (PDOX) nude mouse model with the technique of surgical orthotopic implantation (SOI), including pancreatic [6–9], breast [10], ovarian [11], lung [12], cervical [13], colon [14–16], stomach [17], sarcoma [18–22], and melanoma [23].

The tumor-targeting *Salmonella typhimurium* A1-R (*S. typhimurium* A1-R), developed by our laboratory [24], is auxotrophic for Leu-Arg, which prevents it from mounting a continuous infection in normal tissues. *S. typhimurium* A1-R was effective against primary and metastatic tumors as monotherapy in nude mouse models of major cancers including prostate [25, 26], breast [27–29], lung [30, 31], pancreatic [8, 32–35], ovarian [36, 37] stomach [38], and cervical cancer [39], as well as sarcoma cell lines [40–42] and glioma [43, 44], all of which are highly aggressive tumor models. In addition, *S. typhimurium* A1-R was effective against patient-derived orthotopic models of pancreatic cancer [8, 35], sarcoma [20–22] and melanoma [23].

In a previous study, we used the PDOX model of melanoma to test sensitivity to three molecularly-targeted drugs and one standard chemotherapeutic. A melanoma with a BRAF-V600E mutation was resected from the right chest wall of a patient. The melanoma was grown orthotopically in the right chest wall of nude mice to establish a PDOX model. Trametinib (TRA), a MEK inhibitor caused tumor regression. In contrast, another MEK inhibitor, cobimetinib (COB), slowed but did not arrest growth or cause regression of the melanoma. TEM could slow but not arrest tumor growth or cause regression. Vemrafenib (VEM), which targets the BRAF-V600E mutation would be considered to be a strong candidate for VEM as first-line therapy, was not effective. These results demonstrated the powerful precision of the PDOX model for cancer therapy, not achievable by genomic analysis alone [45].

The combination of *S. typhimurium* A1-R and cisplatinum (CDDP), both at low-dose, also significantly suppressed the growth of another melanoma PDOX with less side effects than high-dose CDDP monotherapy [23].

In the present study, we evaluated the efficacy of *S. typhimurium* A1-R alone and in combination with TEM on a PDOX model for melanoma with the BRAF-V600E mutation.

## RESULTS AND DISCUSSION

**Figure 1:**
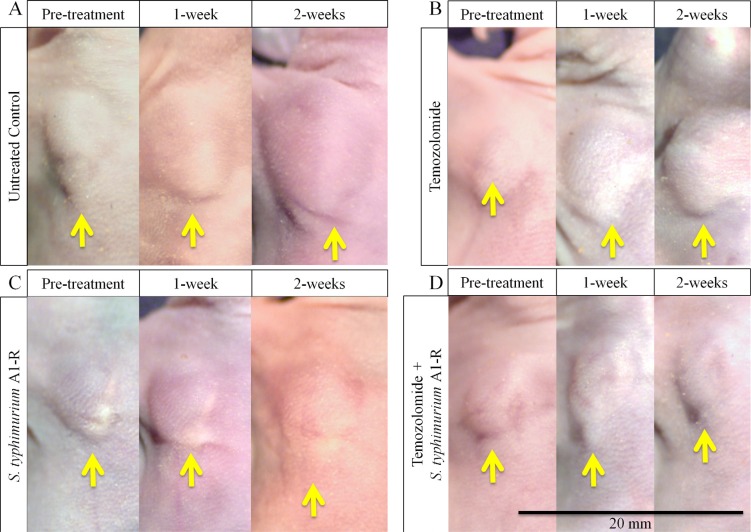
Macroscopic demonstration of therapeutic efficacy of TEM and S. *typhimurium* A1-R on a melanoma PDOX (**A**) Tumor size of the untreated control mice increased over time. (**B, C**) Tumors treated with TEM or *S. typhimurium* A1-R were inhibited. (**D**) Tumors treated with TEM combined with *S. typhimurium* A1-R regressed. Yellow arrows show PDOX tumors on right chest wall. Scale bar: 20 mm.

**Figure 2:**
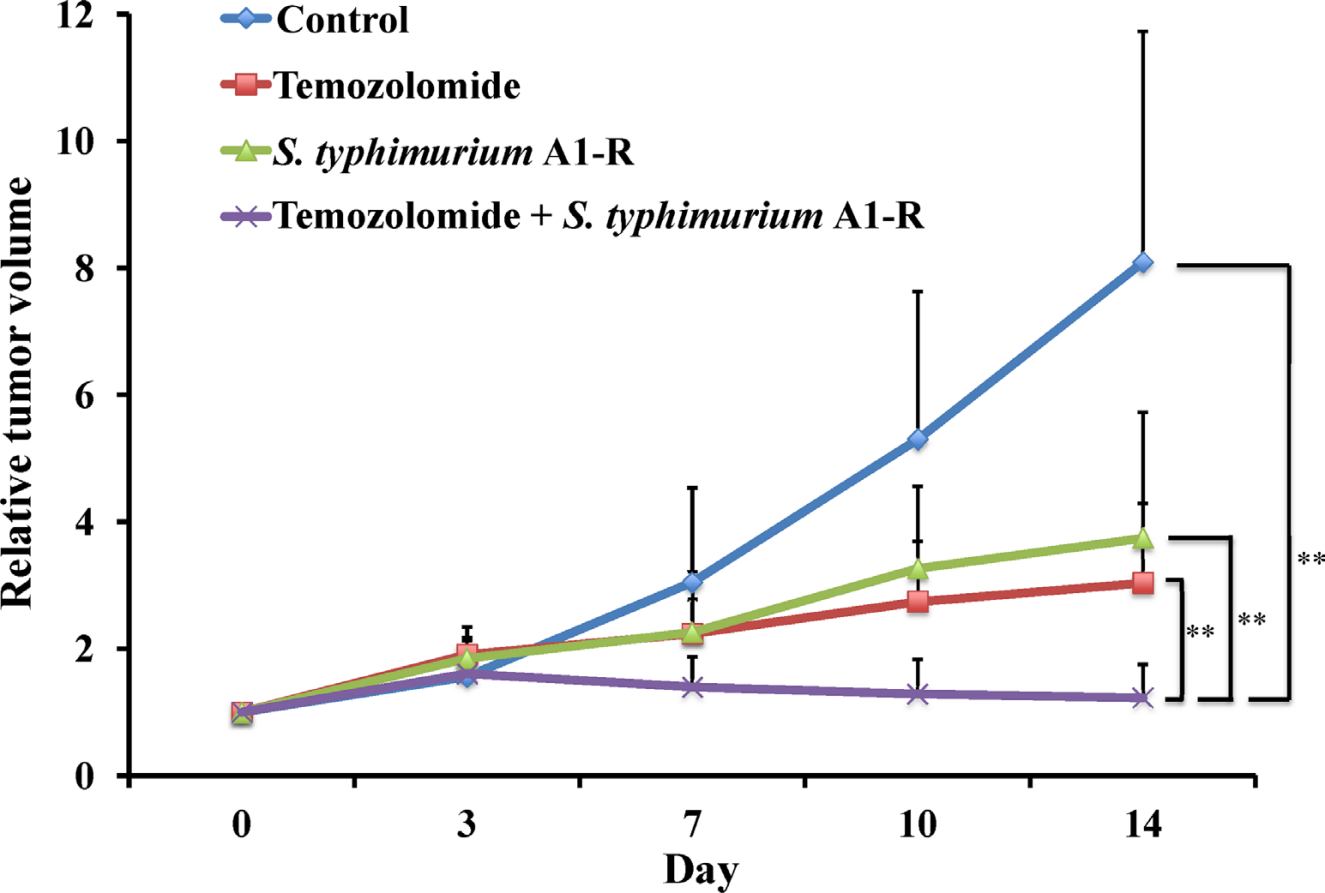
TEM combined with S. *typhimurium* A1-R regressed a melanoma PDOX model Line graph shows relative tumor volume at each point relative to the initial tumor volume. TEM combined with *S. typhimurium* A1-R significantly regressed tumor growth compared to both untreated control and monotherapy of either agent. **p < 0.01. Error bars: ± SD.

**Figure 3:**
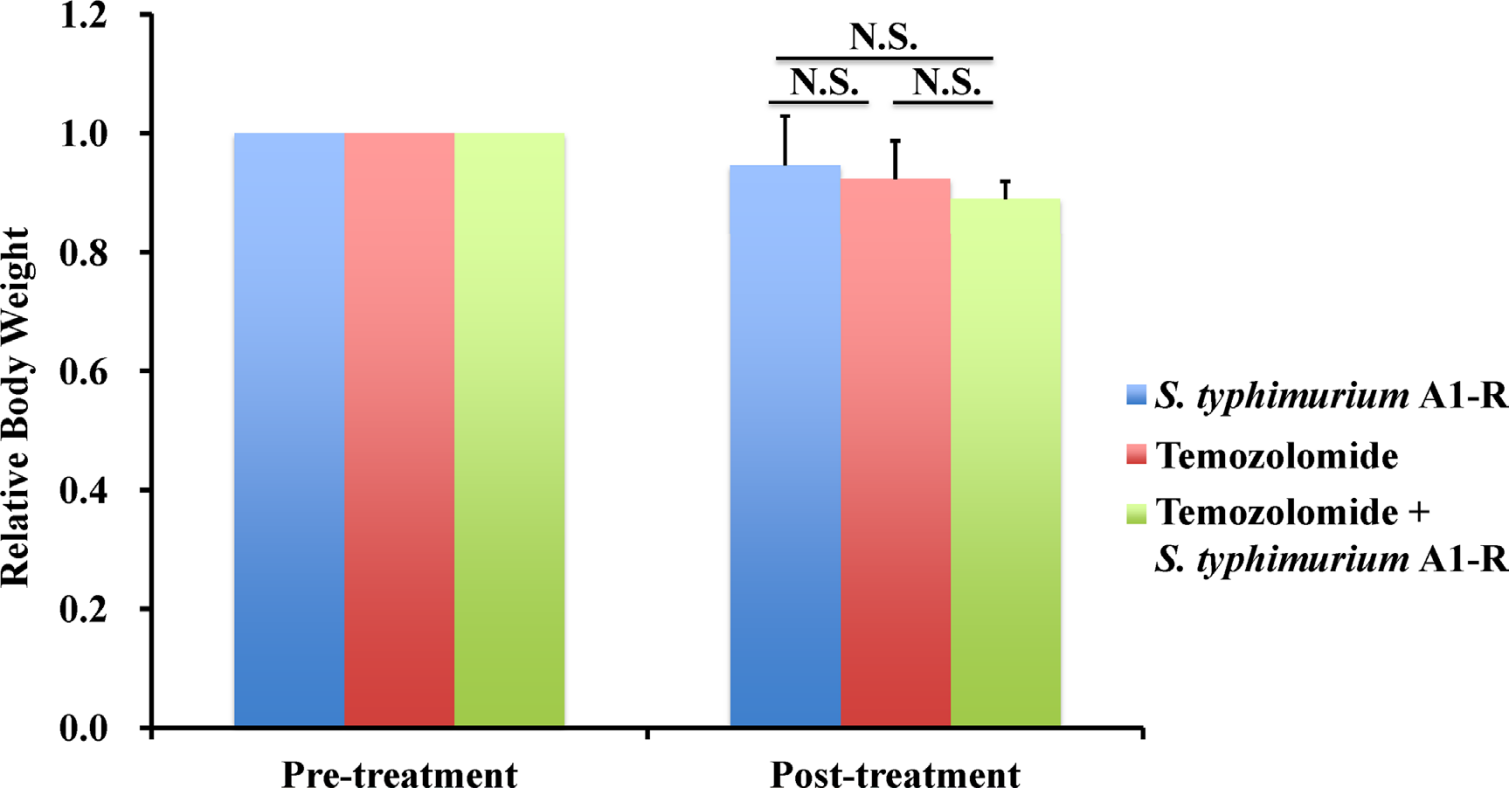
Effect of TEM combined with S. *typhimurium* A1-R on mouse body weight Bar graph shows relative body weight in each treatment group at pre- and post-treatment relative to initial body weight. There were no significant differences between any of the treatment groups and control.

All treatments significantly inhibited tumor growth compared to untreated control (TEM: *p* < 0.0001; *S. typhimurium* A1-R: *p* < 0.0001; TEM combined with *S. typhimurium* A1-R: *p* < 0.0001) on day 14 after initiation. TEM combined with *S. typhimurium* A1-R was significantly more effective than both *S. typhimurium* A1-R (*p* = 0.0004) and TEM alone (*p* = 0.0017) and regressed the tumor. There was no significant difference between the efficacy of *S. typhimurium* A1-R and TEM on the melanoma PDOX (*p* = 0.5205) (Figures 1, 2). The relative body weight on day 14 compared with day 0 did not significantly differ between each treatment group (Figure 3).

**Figure 4:**
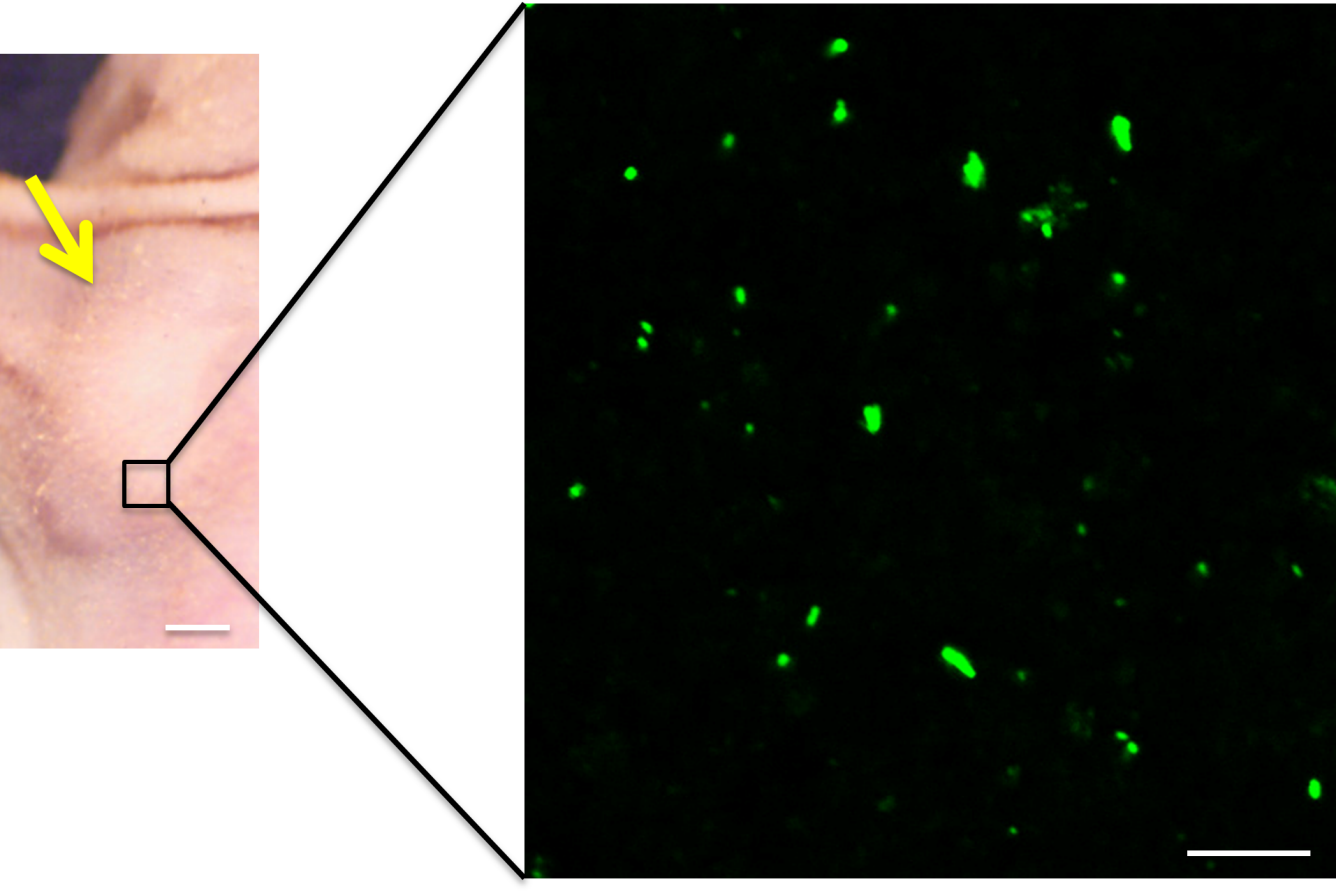
Fluorescence imaging of S. *typhimurium* A1-R-GFP targeting the melanoma PDOX Confocal imaging with the FV1000 demonstrated *S. typhimurium* A1-R-GFP targeting the melanoma PDOX. Bars: left panel: 5 mm, right panel: 12.5 μm.

**Figure 5:**
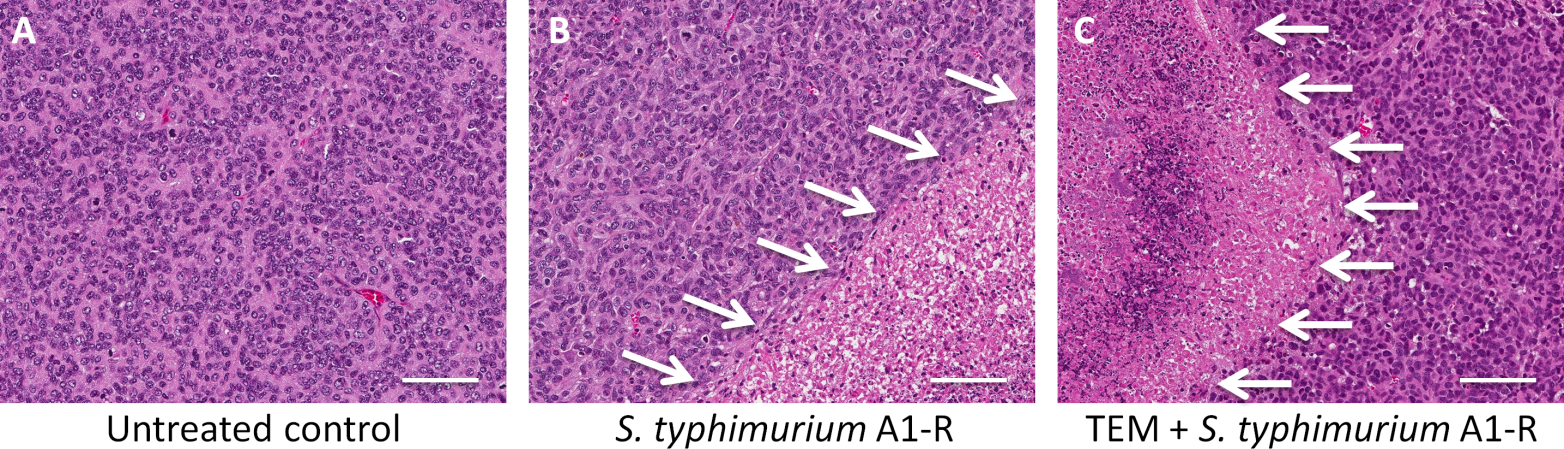
Tumor histology (**A**) Untreated control was comprised of viable cells without obvious necrosis. (**B**) Tumor treated with *S. typhimurium* A1-R had significant necrosis. (**C**) Tumor treated with the combination of TEM and *S. typhimurium* A1-R showed more necrosis. White allows: necrotic areas. Scale bars: 100 μm.

Confocal microscopy showed that the *S. typhimurium* A1-R could directly target the melanoma PDOX (Figure 4) and cause tumor necrosis (Figure 5).

The histology of the original patient tumor and the untreated PDOX tumor were similar, containing the same types of cells. However, nests of cancer cells were seen in the original, but not in the PDOX. Also, the original tumor was slightly melanotic, but the PDOX tumor did not appear to contain melanin [45].

TEM, an alkylating agent, had been widely used as a standard chemotherapy for melanoma. Currently, several molecular targeting agents or immunotherapy are often the first line for melanoma treatment. However, not all melanomas have mutations that are targeted by these new agents and not all patients with these mutations are responsive to these drugs [1].

We previously showed with the present melanoma PDOX that TRA, a MEK inhibitor, was very active and could arrest this tumor, but that COB, another MEK inhibitor, could not arrest the melanoma PDOX. In addition, we showed the VEM was inactive against this melanoma PDOX [45], even though it targets the BRAF-V600E mutation in this melanoma [46].

Despite progress in melanoma therapy, there is still no cure for stage III and IV disease due to drug resistance, tumor heterogeneity and an immunosuppressive tumor microenvironment [1–5]. In addition, the presence of melanin appears to interfere with chemotherapy and radiotherapy of this recalcitrant disease [3]. The present results demonstrate the potential of *S. typhimurium* A1-R to significantly increase the efficacy of first-line melanoma therapy, TEM.

## MATERIALS AND METHODS

### Mice

Athymic nu/nu nude mice (AntiCancer Inc., San Diego, CA), 4–6 weeks old, were used in this study. All mouse surgical procedures and imaging were performed with the animals anesthetized by subcutaneous injection of a ketamine mixture (0.02 ml solution of 20 mg/kg ketamine, 15.2 mg/kg xylazine, and 0.48 mg/kg acepromazine maleate). The response of animals during surgery was monitored to ensure adequate depth of anesthesia. The animals were observed on a daily basis and humanely sacrificed by CO_2_ inhalation if they met the following humane endpoint criteria: severe tumor burden (more than 20 mm in diameter), prostration, significant body weight loss, difficulty breathing, rotational motion and body temperature drop. Animals were housed in a barrier facility on a high efficacy particulate arrestance (HEPA)-filtered rack under standard conditions of 12-hour light/dark cycles. The animals were fed an autoclaved laboratory rodent diet. All animal studies were conducted in accordance with the principles and procedures outlined in the National Institutes of Health Guide for the Care and Use of Animals under Assurance Number A3873-1.

### Patient-derived tumor

A 75-year-old female patient diagnosed with a melanoma of the right chest wall. The tumor was resected in the Department of Surgery, University of California, Los Angeles (UCLA). Written informed consent was provided by the patient, and the Institutional Review Board (IRB) of UCLA approved this experiment [45].

### Establishment of PDOX models of melanoma by surgical orthotopic implantation (SOI)

A fresh sample of the melanoma of the patient was obtained and transported immediately to the laboratory at AntiCancer, Inc., on wet ice. The sample was cut into 5-mm fragments and implanted subcutaneously in nude mice. After three weeks, the subcutaneously-implanted tumors grew to more than 10 mm in diameter. The subcutaneously-grown tumors were then harvested and cut into small fragments (3 mm^3^). After nude mice were anesthetized with the ketamine solution described above, a 5-mm skin incision was made on the right chest into the chest wall, which was split to make space for the melanoma tissue fragment. A single tumor fragment was implanted orthotopically into the space to establish the PDOX model. The wound was closed with a 6–0 nylon suture (Ethilon, Ethicon, Inc., NJ, USA) [45].

### Preparation and administration of *S. typhimurium* A1-R

GFP-expressing *S. typhimurium* A1-R bacteria (AntiCancer Inc.,) were grown overnight on LB medium (Fisher Sci., Hanover Park, IL, USA) and then diluted 1:10 in LB medium. Bacteria were harvested at late-log phase, washed with PBS, and then diluted in PBS. S. typhimurium A1-R was injected intravenously. A total of 5 × 10^7^ CFU *S. typhimurium* A1-R in 100 μl PBS was administered to each mouse [25–27].

### Treatment study design in the PDOX model of melanoma

PDOX mouse models were randomized into four groups of 10 mice each: untreated control (*n* = 10); treated with TEM (25 mg/kg, administrated orally daily for 14 consecutive days, *n* = 10) [1]; treated with *S. typhimurium* A1-R (5 × 10^7^ CFU/100 μl, i.v., once a week for 2 weeks, *n* = 10); treated with TEM (25 mg/kg, administrated orally daily for 14 consecutive days) combined with *S. typhimurium* A1-R (5 × 10^7^ CFU/100 μl, i.v., once a week for 2 weeks, *n* = 10). Tumor length and width were measured twice a week. Tumor volume was calculated with the following formula: Tumor volume (mm^3^) = length (mm) × width (mm) × width (mm) × 1/2. Data are presented as mean ± SD. The tumor volume ratio is defined at the tumor volume at any given time point relative to the initial tumor volume.

### Confocal microscopy

The FV1000 confocal microscope (Olympus, Tokyo, Japan) was used for high-resolution imaging. Fluorescence images were obtained using the 20×/0.50 UPlan FLN and 40×/1.3 oil Olympus UPLAN FLN objectives [47].

### Histological examination

Fresh tumor samples were fixed in 10% formalin and embedded in paraffin before sectioning and staining. Tissue sections (5 μm) were deparaffinized in xylene and rehydrated in an ethanol series. Hematoxylin and eosin (H&E) staining was performed according to standard protocols. Histological examination was performed with a BHS System Microscope (Olympus Corporation, Tokyo, Japan). Images were acquired with INFINITY ANALYZE software (Lumenera Corporation, Ottawa, Canada) [20].

### Statistical analysis

JMP version 11.0 was used for all statistical analyses. Significant differences for continuous variables were determined using the Mann-Whitney *U* test. Line graphs expressed average values and error bar showed SD. A probability value of *P* ≤ 0.05 was considered statistically significant.

## CONCLUSIONS

The combination of TEM and *S. typhimurium* A1-R was more effective than each mono-therapy for melanoma in a PDOX mouse model and could regress the tumor. This treatment strategy has important future clinical application, which possibly can be realized in the near future.

Previously-developed concepts and strategies of highly-selective tumor targeting can take advantage of molecular targeting of tumors, including tissue-selective therapy which focuses on unique differences between normal and tumor tissues [48–53].
